# Algal Toxins, Food Safety, and Bioterrorism: A Food and Water Defense Perspective

**DOI:** 10.3390/toxics14090779

**Published:** 2026-08-31

**Authors:** Antonello Novelli, Maria Teresa Fernández-Sánchez, Ann M. Marini, Peter Pressman, A. Wallace Hayes

**Affiliations:** 1 University Institute of Biotechnology of Asturias (IUBA), University of Oviedo, 33006 Oviedo, Spain; antonello9lli@gmail.com; 2 Department of Psychology, University of Oviedo, 33003 Oviedo, Spain; 3 Institute for Health Research of the Principality of Asturias (ISPA), 33011 Oviedo, Spain; 4 Department of Biochemistry and Molecular Biology, University of Oviedo, 33006 Oviedo, Spain; mfernandez@uniovi.es; 5 Department of Neurology, Programs in Neuroscience, Molecular and Cellular Biology, Graduate School of Nursing, Uniformed Services University of the Health Sciences, Bethesda, MD 20814, USA; ann.marini@usuhs.edu; 6 Department of Social and Medical Science, University of Maine, Orono, ME 04469, USA; drpressvm2@gmail.com; 7 College of Public Health, University of South Florida, Tampa, FL 32610, USA

**Keywords:** algal toxins, harmful algal blooms, food defense, water security, cyanotoxins, seafood safety, drinking water, emergency preparedness

## Abstract

Food defense encompasses both the prevention of accidental contamination and the protection of food and water systems against deliberate adulteration. Within this framework, algal toxins deserve greater attention because they occupy a critical intersection of water security, seafood and drinking-water safety, environmental change, supply-chain continuity, and public health preparedness. The novelty of this review lies in reframing algal toxins from a primarily environmental, seafood safety, or clinical toxicology topic into an integrated food and water defense problem. This perspective brings together harmful algal bloom ecology, toxin toxicology, seafood safety, drinking-water protection, preparedness for intentional adulteration, climate change risk, surveillance, and emergency response. Algal toxins can enter the human supply chain through multiple marine and freshwater pathways, are often undetectable by taste, odor, or appearance, may resist routine preparation measures, and can generate high-consequence disruptions even when contamination begins locally. The analysis further shows that prevention depends largely on upstream measures—including environmental surveillance, harvest-area restrictions, source-water protection, testing, supply-chain controls, and rapid public communication—rather than on consumer behavior. Framed in the context of the U.S. Food and Drug Administration’s Intentional Adulteration Rule under the Food Safety Modernization Act, this paper situates algal toxins within a preventive defense model that integrates monitoring, vulnerability reduction, and emergency preparedness. Although most harmful algal bloom events are naturally occurring, algal toxins warrant attention in food-defense planning because they can contaminate seafood and drinking-water systems, complicate detection and attribution, and expose vulnerabilities across interconnected food and water infrastructures.

## 1. Introduction

**Figure 1:**
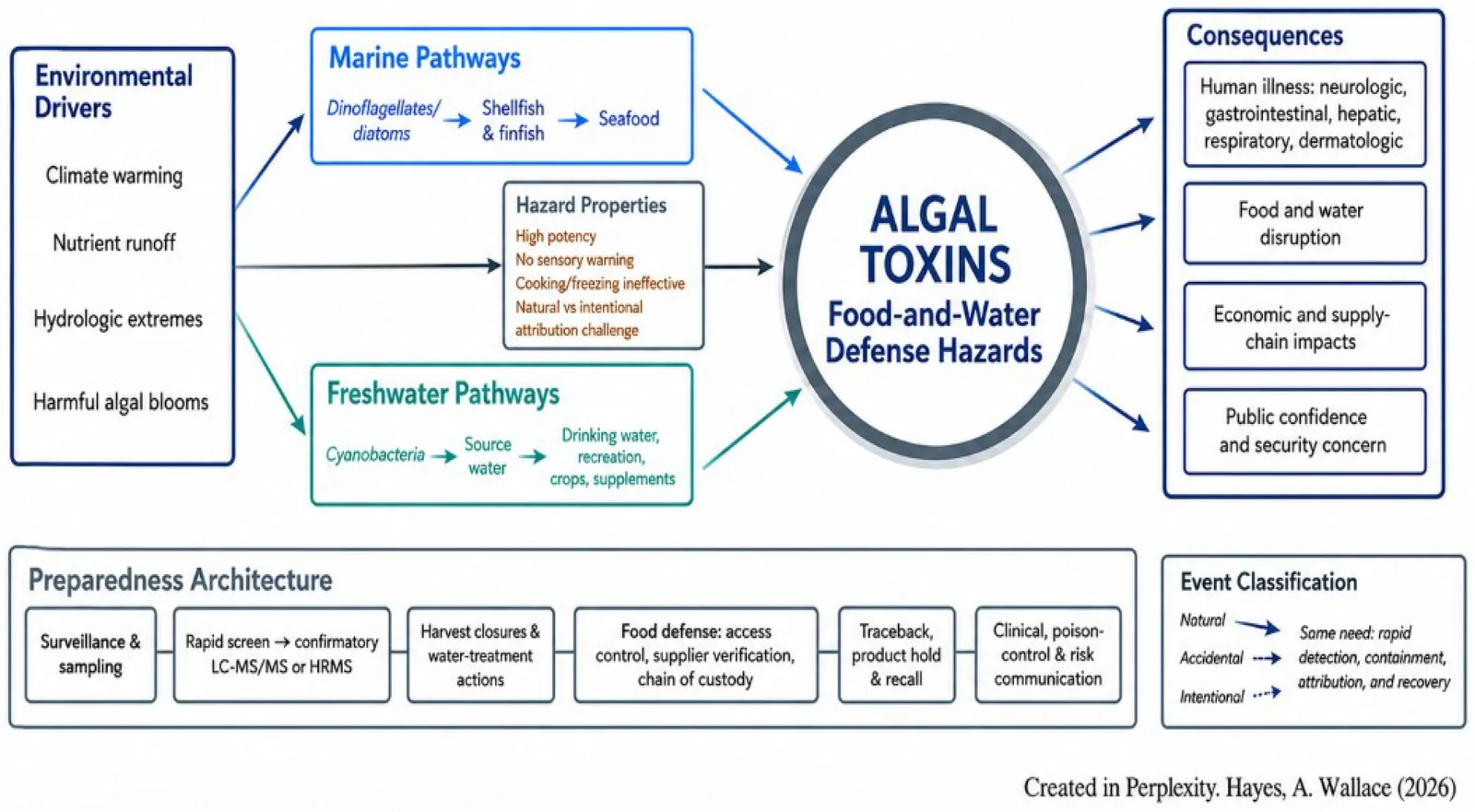
Schematic Diagram of A Potential Food/Water Defense For Algal Toxins.

The rationale for treating algal toxins as a food and water defense issue begins with their multiple routes of entry into human food and water systems [Figure 1]. In marine environments, filter-feeding shellfish—including oysters, clams, and mussels—can bioaccumulate toxins produced by toxic organisms, while some finfish acquire them through trophic transfer. These exposure pathways are linked to recognized seafood poisoning syndromes, including paralytic shellfish poisoning caused by saxitoxins, amnesic shellfish poisoning caused by domoic acid, diarrhetic shellfish poisoning caused by okadaic acid and dinophysistoxins, neurotoxic shellfish poisoning caused by brevetoxins, azaspiracid shellfish poisoning, and ciguatera fish poisoning caused by ciguatoxins [1,2]. In freshwater systems, cyanotoxins such as microcystins, cylindrospermopsin, anatoxin-a, and saxitoxins may contaminate drinking-water sources, recreational waters, fish, irrigated crops, and blue-green algae dietary supplements [2,3]. Collectively, these pathways show that algal toxins are not confined to isolated ecological settings; they can move through interconnected food, water, and consumer-product systems, complicating prevention, detection, and response.

Publicly documented cases of deliberate, population-scale contamination with algae, cyanobacteria, or their toxins are rare; however, historical weapons programs demonstrate intentional interest in algal-derived toxins, particularly saxitoxin, which was retained by the US Central Intelligence Agency for possible operational use and remains regulated under the Chemical Weapons Convention and US select agent rules [4,5,6,7]. Natural harmful algal bloom events provide useful consequence analogs: during the 2014 Toledo, Ohio drinking-water crisis, microcystin contamination prompted a do-not-drink advisory affecting more than 440,000 residents and caused substantial health, economic, and public-confidence impacts [8]. From a threat-assessment perspective, deliberate introduction of purified toxin or toxigenic material into a localized food or water system may be more plausible than inducing a sustained bloom, because bloom establishment and toxin production depend on variable ecological conditions; overall likelihood is therefore generally low, but localized consequences and psychological disruption could be high [2,5,6,7]. Security measures should include source-to-tap monitoring, secure custody of toxin standards and cultures, protection of treatment and sampling infrastructure, confirmatory laboratory testing, coordinated public health and law enforcement response, and clear risk communication [7,9,10,11]. Because cyanotoxins may arise naturally, attribution should integrate analytical toxin profiles, ecological and hydrological evidence, operational records, chain-of-custody documentation, and indications of tampering rather than treating toxin detection alone as proof of malicious intent [9,10,11].

Their defense relevance is heightened because many algal toxins are not detectable by consumers through taste, odor, or appearance. The Centers for Disease Control and Prevention (CDCs) guidance notes that algal toxins in fish and shellfish may be organoleptically undetectable and that cooking, freezing, or preservation do not reliably remove them [12]. Similarly, boiling water contaminated with cyanotoxins does not ensure safety and may concentrate toxins as water evaporates [13]. These properties shift risk management away from consumer-level safeguards and toward upstream controls, including environmental monitoring, harvest-area closures, product testing, source-water management, treatment optimization, and rapid public notification. The central defense problem is therefore not simply exposure, but the limited ability of routine consumer behaviors to interrupt exposure once contamination has entered food or water systems.

This logic explains why algal toxins are relevant to bioterrorism and intentional contamination planning, even though most harmful algal bloom events are naturally occurring. Their significance does not rest on evidence of frequent deliberate use, but on features central to defense analysis: biological origin, high potency, potential effects on widely consumed foods and water supplies, absence of obvious sensory warning, and the difficulty of distinguishing intentional contamination from naturally occurring events during the early phase of an incident. Saxitoxin, among other algal toxins, is particularly important because it is both a naturally occurring algal toxin associated with paralytic shellfish poisoning and a select harmful algal bloom (HAB) toxin identified by the Federal Select Agent Program as capable of posing a threat to public health and safety [14,15]. This designation underscores a broader preparedness principle: food-defense systems must be able to detect, contain, and manage high-consequence toxin hazards regardless of whether an event is ultimately classified as environmental, accidental, or intentional because of rapid, uncontrolled overgrowths of microscopic algae or cyanobacteria (blue-green algae) that can release toxins into marine and freshwater environments [16].

The contribution of this review is not to suggest that algal toxins are commonly used as deliberate agents, but to explain why they should be incorporated into the preparedness architecture used for other high-consequence food and water hazards. The existing harmful algal bloom literature often treats bloom ecology, seafood poisoning, drinking-water management, and clinical toxicology as separate domains. A food and water defense perspective integrates these domains around operational questions that determine incident outcomes: where contamination can enter or concentrate, how rapidly it can be detected, which products or water systems may be affected, how exposure can be interrupted, and how public health authorities can communicate credible risk information before preventable illness occurs.

Table 1 summarizes the principal algal toxin classes discussed in this review, with emphasis on representative toxins, source organisms, and dominant molecular targets relevant to human health [17].

The broader significance of algal toxins becomes clearer when viewed against the scale and interdependence of contemporary food and water systems. The World Health Organization estimates that unsafe food causes approximately 600 million illnesses and 420,000 deaths globally each year and emphasizes that increasingly globalized food chains can allow local contamination events to become international concerns [33]. In the United States, commonly cited federal estimates attribute roughly 48 million foodborne illnesses, 128,000 hospitalizations, and 3000 deaths annually to foodborne hazards overall [34]. Although these figures are not specific to algal toxins, they illustrate the systemic context in which toxin-mediated hazards operate: localized environmental contamination can become a supply-chain, healthcare, and public trust crisis when it intersects with centralized processing, interstate commerce, or essential water infrastructure. For that reason, algal toxin hazards should be incorporated into preventive food safety and defense systems rather than treated solely as episodic environmental anomalies.

Seafood safety programs are central to preventing exposure to algal toxins. The National Shellfish-Sanitation Program protects public health for bivalve molluscan shellfish in interstate commerce through cooperation among state agencies, the FDA, the EPA, NOAA, and the shellfish industry [35,36]. This cooperative structure is important because shellfish can concentrate marine biotoxins even when the surrounding water appears safe to consumers. State officials may close harvest areas when monitoring detects unsafe levels of toxic algae or shellfish toxins. Consumers are advised to follow local shellfish, fishing, and swimming advisories [35,37].

Comparable protection at the international level is provided through a layered system of technical guidance, food standards, scientific risk assessment, harmful algal bloom surveillance, and national enforcement. WHO and FAO jointly publish technical guidance for developing the growing area components of bivalve mollusk sanitation programs, while the FAO/IOC/WHO assessment of biotoxin risks supplies scientific information for risk assessment, monitoring, surveillance, analytical testing, and public health management [38,39]. Codex Alimentarius, the joint FAO/WHO food standards program, is not itself an enforcement agency, but its standards provide a harmonized foundation for national controls. The Codex standards for live and raw bivalve molluscs and for contaminants and toxins in food specify maximum levels for major marine-biotoxin groups, including the saxitoxin, okadaic acid, domoic acid, brevetoxin, and azaspiracid groups [40,41]. In Europe, the European Food Safety Authority’s Panel on Contaminants in the Food Chain evaluates toxicity, acute dietary exposure, analytical methods, and the adequacy of regulatory limits for established and emerging marine biotoxins. EFSA provides scientific risk assessments rather than operating harvest-area monitoring; regulatory and enforcement actions are undertaken by European Union institutions and national authorities [42].

IOC-UNESCO’s International Harmful Algal Bloom (HAB) Program and the IOC-FAO Intergovernmental Panel on Harmful Algal Blooms strengthen the ability of member states to understand, predict, monitor, and mitigate harmful algal blooms. Their activities include training in harmful microalgae identification, toxicity testing, monitoring methods, and management strategies [43]. The IOC-supported Harmful Algal Information System and Harmful Algae Event Database provide an international mechanism for documenting and comparing harmful algal events and their effects on public health, fisheries, aquaculture, and coastal communities [44]. These international efforts were further strengthened in 2026 by joint FAO/IOC-UNESCO/IAEA guidance covering the sampling and analysis of algal toxins in bivalve mollusks, monitoring of toxic microalgae, and management of harvesting and production areas [45].

In Asia, comparable protection is delivered through overlapping regional networks and country-level surveillance and control systems rather than through one harmonized shellfish-sanitation program. The IOC-WESTPAC Harmful Algal Blooms Program coordinates information exchange among Western Pacific countries and conducts training and capacity-building on ecology, identification, toxicity, and detection of harmful algae [46]. The North Pacific Marine Science Organization’s Section on Ecology of Harmful Algal Blooms, or PICES S-HAB, supports the exchange of national occurrence data, scientific findings, and monitoring approaches among North Pacific countries and contributes information to international HAB databases [47]. ASEAN’s broader Food Safety Policy promotes Codex-based harmonization, risk-based national food control systems, traceability, food recall procedures, and information exchange through the ASEAN Rapid Alert System for Food and Feed. In parallel, the Southeast Asian Fisheries Development Center’s Marine Fisheries Research Department has conducted regional projects and training to improve the monitoring, laboratory detection, and identification of seafood biotoxins and biotoxin-producing HAB species in ASEAN countries [48,49].

National programs translate these international and regional frameworks into operational controls. Japan combines prefectural monitoring of harmful phytoplankton with regular shellfish toxin testing; exceedances result in voluntary shipment restrictions, harvest restraint, and public warnings, and shipment generally resumes only after toxin concentrations remain below the regulatory level for three consecutive weeks [50]. The Republic of Korea conducts year-round shellfish toxin surveillance, intensifies sampling during higher-risk seasons, designates harvest-prohibited areas when limits are exceeded, requires pre-shipment testing from affected areas, and communicates results to fishers and the public [51]. In the Philippines, the Bureau of Fisheries and Aquatic Resources issues recurring Shellfish Bulletins that identify areas where paralytic shellfish toxins exceed regulatory limits and advise the public not to harvest, sell, purchase, or consume shellfish from affected waters [52]. Together, these programs form a multilayered prevention system that connects international standards and risk assessment with regional scientific cooperation and local monitoring, testing, harvest restrictions, traceability, and public risk communication.

*CXS 292-2008* is listed as a Schedule 1 toxic chemical under the Chemical Weapons Convention (CWC), seafood-toxin programs must comply with chemical-security requirements when producing, possessing, using, or transferring purified toxin standards or concentrated preparations. Schedule 1 status does not make naturally occurring saxitoxin in harmful algal blooms or contaminated shellfish a chemical weapon; under the CWC’s general-purpose criterion, legal status depends on the intended purpose and whether the type and quantity involved are consistent with that purpose [6,53]. Shellfish monitoring, diagnostic testing, analytical method validation, medical research, and other legitimate public health activities are permitted, but purified saxitoxin may be produced, acquired, retained, or used only for research, medical, pharmaceutical, or protective purposes and in accordance with national implementing legislation and oversight by the relevant CWC National Authority [53,54,55,56]. International transfers are limited to CWC State Parties; retransfer to a third state is prohibited, and transfers normally require notification to the OPCW at least 30 days in advance. However, transfers of 5 mg or less for medical or diagnostic purposes may be notified at the time of transfer, although annual declaration and other control requirements continue to apply [53,54,55]. This limited exception was introduced because advance-notification requirements had delayed access to calibration standards and diagnostic materials needed for paralytic shellfish poisoning surveillance [55]. The CWC therefore complements, rather than replaces, WHO, FAO, Codex Alimentarius, IOC-UNESCO, regional harmful algal bloom programs, and national shellfish-sanitation systems by adding controls for security, traceability, international movement, and the prevention or investigation of deliberate misuse of saxitoxin [53,56].

Tetrodotoxin (TTX) is a potent, non-protein neurotoxin best known from pufferfish but also detected in other marine and terrestrial organisms. Its toxicological importance derives from its high-affinity blockade of tetrodotoxin-sensitive voltage-gated sodium channels, which prevents the sodium influx required for action-potential initiation and propagation, and can cause paresthesia, weakness, flaccid paralysis, and respiratory failure, while consciousness may initially be preserved [57,58,59].

From a biological-security perspective, TTX is best characterized as a toxin threat rather than a conventional infectious biological agent: it cannot replicate, does not cause communicable disease, and cannot produce secondary transmission. Nevertheless, toxin-focused bioterrorism reviews include tetrodotoxin among agents of concern because sodium channel inhibition can produce severe neurological poisoning [60]. This concern is tempered by practical constraints: unlike infectious agents, TTX cannot amplify in a population, and large-scale effects would depend on successful acquisition, formulation, delivery, and exposure.

The best documented large exposures are accidental foodborne outbreaks rather than deliberate attacks. In Bangladesh, seven reported pufferfish-poisoning outbreaks from 1998 to June 2008 involved 156 people and 40 deaths, a case-fatality rate of about 26% [61]. More broadly, seafood-associated TTX poisoning has been concentrated in Asia, although TTX and its analogs have also emerged as food safety concerns in European bivalves and gastropods; a global retrospective study identified 3032 seafood-associated human cases reported by April 2018, with most cases in Asia [62,63].

The accessible clinical and public health literature does not establish a pattern of intentional, large-scale TTX attacks. Accordingly, TTX is more appropriately framed as a high-consequence foodborne or targeted-poisoning hazard than as a historically demonstrated mass-casualty weapon. Because no routinely used toxin-specific antidote exists, management is primarily supportive, especially maintenance of ventilation and cardiovascular support until toxin effects resolve [57,59].

The same pharmacology that makes TTX dangerous has also made it scientifically valuable. Selective inhibition of sodium channel subtypes has supported electrophysiology and neuroscience research, and controlled pharmacological applications have been investigated for analgesia and other therapeutic indications [57,58,64,65]. Overall, preparedness should emphasize rapid clinical recognition, analytical detection, supportive critical care, and surveillance for unusual clusters of poisoning.

Drinking-water systems require comparable attention. Cyanobacterial toxins are among the hazardous substances commonly found in water bodies, and the WHO emphasizes that managing lakes, reservoirs, and rivers is critical to preventing cyanobacterial blooms and protecting public health [66]. EPA similarly notes that harmful algal blooms that produce cyanotoxins can threaten drinking-water supplies and that severe bloom events require proactive planning and active management to reduce risk in potable water systems [67,68]. EPA has also issued drinking-water health advisories for microcystins and cylindrospermopsin. Although these advisories are not legally enforceable federal standards, they provide technical guidance for protecting public health during contamination events [29]. Table 2 contrasts marine and freshwater toxin systems to illustrate how environmental setting, exposure pathway, and physicochemical behavior shape public health risk [69].

Water-system preparedness is also a security issue. Under America’s Water Infrastructure Act, community drinking-water systems serving more than 3300 people must develop or update risk and resilience assessments and emergency response plans [74]. These plans are directly relevant to algal toxins because cyanotoxin contamination can affect source-water quality, treatment decisions, public notification, alternative water supplies, healthcare preparedness, and public trust. Together with seafood controls, these water-system requirements clarify why algal toxins belong within a broader food and water defense framework rather than solely within routine environmental monitoring or public health response.

From a food-defense perspective, algal toxins are concerning not only because they can contaminate seafood and other food inputs, but also because they challenge conventional assumptions about control. A vulnerability-based approach asks where an attacker—or a system failure—could introduce, concentrate, misclassify, or distribute toxin-contaminated material to achieve the greatest public health impact. In practice, this means focusing on high-risk points such as harvest-area verification, raw-material approval, receiving, bulk liquid handling, storage, blending, labeling, and distribution. Under the FDA’s food-defense framework, facilities are expected to identify significant vulnerabilities, establish mitigation strategies at actionable process steps, monitor those controls, and verify that they function as intended. For algal toxin hazards, this perspective supports restricted access, chain-of-custody controls, supplier verification, product-hold procedures, and rapid traceback capability for seafood, algal ingredients, and water-dependent foods [3,75].

Table 3 outlines the major analytical and regulatory approaches used to detect, evaluate, and manage algal toxin risk.

From a water-defense perspective, algal toxins demonstrate that source-water protection and treatment resilience are inseparable from security planning. America’s Water Infrastructure Act requires many community water systems to assess risks to source water, treatment processes, storage, distribution, monitoring practices, and emergency response capacity. These requirements align closely with cyanotoxin preparedness. A defensible strategy includes source-water surveillance, clear trigger points for intensified sampling, treatment adjustments, operational redundancy, alternative water planning, and communication protocols that can be activated quickly if finished water is threatened. Because cyanotoxin incidents can resemble naturally occurring blooms yet still cause high-consequence disruptions, utilities also need detection, decision-making, and incident-command systems that enable rapid coordination with public health agencies, laboratories, and emergency managers [12,14,33,74].

## 2. Food and Water Defense Implications

These considerations lead to a clear defense conclusion: algal toxins are not merely environmental contaminants or episodic public health curiosities, but operational hazards that expose vulnerabilities in both food and water systems. In food systems, this means protecting points where contaminated seafood, algal ingredients, or process water could be introduced, concentrated, mislabeled, blended, or distributed at scale. In water systems, it means treating cyanotoxin events not only as water quality incidents but also as disruptions that can affect treatment performance, continuity of service, public communication, healthcare demand, and institutional trust. Across both domains, the same principle applies: effective prevention depends on identifying high-consequence failure points before exposure occurs and integrating monitoring, access control, traceback, emergency planning, and public health response within a single preparedness architecture.

Operationalizing that framework requires an integrated approach that combines food safety, water safety, environmental monitoring, and emergency planning. In practice, this includes monitoring harvest and source waters, testing when indicated, optimizing treatment and control measures, maintaining traceback and communication systems, and applying food-defense safeguards at high-risk points such as sourcing, receiving, processing, storage, blending, and water inputs [3]. Recent international guidance reinforces this operational approach. The 2026 joint FAO/IOC-UNESCO/IAEA guidance on algal toxins in bivalve mollusks emphasizes harmful algae monitoring, management of harvesting and production areas, pre-harvest monitoring, post-harvest batch testing, and microalgal monitoring as components of a risk-based system to prevent toxin-contaminated shellfish from entering commerce [29].

### 2.1. Significance of the Review

The significance of this review lies in explicitly reframing algal toxins as food and water defense hazards rather than treating them only as environmental contaminants, seafood syndromes, or drinking-water quality problems. This framing is important because algal toxins occupy an unusual position among defense-relevant hazards: they are naturally occurring and biologically derived, yet their potency, concealment, resistance to routine consumer mitigation, uncertain attribution, and capacity to disrupt high-volume food and water systems create challenges similar to those considered in intentional contamination planning. The defense question is therefore not limited to whether contamination is deliberate. It is whether the food and water system can rapidly detect, contain, trace, communicate, and recover from high-consequence toxin events before avoidable exposures occur.

This integrated framing also clarifies the practical value of the review. It brings together topics that are often managed separately—harmful algal bloom ecology, toxin mechanisms, seafood controls, drinking-water resilience, clinical recognition, laboratory testing, climate change, intentional adulteration preparedness, and emergency communication—and organizes them around operational vulnerability. That approach supports a preparedness model in which seafood authorities, water utilities, laboratories, poison centers, clinicians, emergency managers, and food-sector operators can plan from a shared risk architecture rather than responding through disconnected programs.

### 2.2. Surveillance and Clinical Recognition

Harmful algal bloom-related illnesses may present with gastrointestinal, neurologic, respiratory, dermatologic, or hepatic effects, depending on the toxin and route of exposure. The CDC notes that treatment for cyanotoxin-associated illnesses and foodborne illnesses caused by harmful algal bloom toxins is generally supportive and symptom-directed, and that no specific antidotes exist for many of these toxins [12]. This reality increases the importance of prevention, early reporting, poison-control consultation, public health notification, and laboratory coordination when contaminated seafood, drinking water, or algal products are suspected.

Recent U.S. outbreak surveillance reinforces the operational importance of this prevention-first approach. During 2011–2023, CDC identified 402 foodborne disease outbreaks associated with marine toxins, resulting in 1280 illnesses, 96 hospitalizations, and one death; ciguatoxin alone caused 189 outbreaks, 619 illnesses, and 67 hospitalizations [86]. Although the CDC marine toxin category also includes non-algal etiologies such as scombroid toxin, the report is directly relevant to algal toxin preparedness because it emphasizes that ciguatoxin and shellfish-associated algal toxins are tasteless, odorless, and resistant to cooking or freezing once aquatic animals are contaminated [86]. These features reinforce the core defense argument of this review: consumer-level detection and routine preparation cannot be relied on as the final safety barrier. The range of human illnesses associated with these toxins is summarized in Table 4 to link exposure pathways to the syndromes most relevant to clinical and public health responses.

### 2.3. Analytical Methodologies for Algal Toxins in Food Defense Preparedness

Robust analytical methods for algal toxins are a core element of food-defense preparedness because harmful algal bloom toxins can contaminate seafood, algal supplements, and drinking-water sources and could plausibly be involved in intentional contamination scenarios. Current best practice emphasizes validated liquid chromatography–tandem mass spectrometry (LC-MS/MS) methods for regulatory confirmation, especially for lipophilic marine biotoxins under EU methodology. Newer multi-class LC-MS/MS workflows extend coverage to cyanotoxins such as microcystins, nodularin, cylindrospermopsin, anatoxin-a, okadaic acid, and domoic acid in water, shellfish, and algal-derived products [97,98,99,100,101,102,103,104]. Solid-phase extraction, matrix-matched calibration, and isotopically labeled internal standards improve sensitivity and compensate for extraction losses and matrix effects, supporting detection at environmentally and food safety-relevant concentrations [99,101,103].

High-resolution mass spectrometry (HRMS) has become increasingly important because it supports both targeted quantification and untargeted or retrospective screening for emerging toxin analogs, transformation products, and unusual contamination profiles that may not be captured by narrow targeted assays [104,105]. However, HRMS and other advanced screening tools still require rigorous validation, including assessments of specificity, linearity, accuracy, precision, limits of detection and quantification, measurement uncertainty, and interlaboratory comparability. Quality assurance frameworks from the FDA, European standards, and related guidance emphasize the use of certified reference materials where available, confirmatory analysis, and documented performance criteria to ensure defensible results during routine monitoring and emergency response [100,106,107].

Rapid screening methods, including enzyme-linked immunosorbent assays, lateral-flow devices, electrochemical biosensors, and portable field platforms, are valuable for triage because they can provide faster preliminary results for toxins such as saxitoxins, domoic acid, microcystins/nodularins, and anatoxin-a [108,109]. These approaches are best used as part of a tiered strategy: rapid tests identify samples requiring urgent action, while LC-MS/MS or HRMS provides confirmatory identification and quantification. International coordination by FAO, IAEA, IOC UNESCO, Codex-related programs, and national regulators is increasingly focused on harmonized monitoring, sampling, classification, and testing guidance so that laboratories can respond consistently to natural harmful algal bloom events as well as food-defense incidents [110,111].

### 2.4. Climate Change and Emerging Risks

Climate change is best understood as both a driver of harmful algal bloom dynamics and a threat multiplier for food and water defense. Warmer surface waters, altered precipitation, intensified stratification, nutrient runoff, eutrophication, drought–flood cycles, marine heatwaves, freshening, sea-level rise, and changing circulation patterns can increase bloom frequency, prolong bloom duration, expand geographic range, and shift species composition across freshwater, estuarine, and marine systems [112,113,114,115]. These environmental changes are operationally important because the risk posed by harmful algal blooms depends not only on whether blooms occur, but also on which organisms dominate, what toxins they produce, how toxins move through seafood, drinking water, recreational, wildlife, and subsistence pathways, and whether surveillance systems can detect changing exposure profiles before preventable illness or disruption occurs.

Recent evidence supports this integrated risk model. A 2024 review in Nature Reviews Earth and Environment reported that global inland harmful algal bloom occurrence has increased since the 1980s, including a 44% increase from the 2000s to the 2010s, and identified nutrient pollution, climate warming, legacy nutrients, integrated monitoring networks, and data-sharing frameworks as central to forecasting and mitigation [116]. In high-latitude coastal systems, modeling and observational studies indicate that warming and freshening may alter bloom frequency, seasonality, and toxin profiles. Along the Norwegian coast, a 3 °C warmer scenario was projected to increase Dinophysis acuta bloom frequency while reducing Alexandrium tamarense-complex blooms, implying a possible shift in relative exposure from paralytic to diarrhetic shellfish toxins in some settings [117].

Field studies further show that climate-related ocean change can translate into food web exposure. A 2025 Nature study quantified domoic acid and saxitoxin in bowhead whale samples collected over 19 years and found that toxin prevalence and concentrations were correlated with ocean heat flux, open-water area, wind velocity, and atmospheric pressure, supporting a mechanistic link between warming conditions and increasing algal toxin concentrations in Arctic food webs [118]. These findings are relevant to food and water defense because they link environmental change to seafood safety, subsistence harvesting, wildlife health, food security, and the need for surveillance systems that can detect northward, seasonal, and species-specific shifts in toxin risk.

A One Health perspective reinforces this conclusion by showing that algal toxins can move across ecological, animal, and human systems during climate-sensitive events. A 2026 study of a harmful algal bloom in Golfo Nuevo, Argentina documented phycotoxin transfer from phytoplankton to mesozooplankton, mussels, fish, whales, and sea lions; it also described sea lion mortality, evidence of maternal toxin transfer, and gastrointestinal illness in approximately 10% of the local population during the bloom period [119]. In the Arctic, rapid detection and risk communication during a large *Alexandrium catenella* bloom demonstrated the value of near-real-time monitoring, interdisciplinary coordination, and stakeholder engagement in remote regions where routine laboratory capacity and public health infrastructure may be limited [120]. Together, these studies show that emerging climate-driven algal toxin risks are not confined to environmental monitoring; they affect seafood safety, public communication, emergency preparedness, wildlife conservation, subsistence food security, and confidence in food and water systems.

The same climate pressures can also weaken the infrastructure on which food-defense controls depend. Extreme heat, floods, drought, wildfire, sea-level rise, and severe storms can disrupt electricity, refrigeration, potable water supplies, transportation, communications, automated controls, cybersecurity, and physical security throughout food production, processing, storage, and distribution [121,122,123]. Emergency conditions may require temporary workers, alternative suppliers, manual operations, mobile equipment, or new water sources, thereby increasing opportunities for unauthorized access and reducing the reliability of established mitigation measures. These effects connect climate resilience with federal food and water defense requirements: the FDA Intentional Adulteration Rule requires periodic vulnerability reassessment for covered facilities, while the America’s Water Infrastructure Act requires community water systems serving more than 3300 people to assess both natural hazards and malevolent acts and to update risk and emergency response plans at least every five years [121,124]. Climate-adjusted assessments should therefore evaluate how critical process steps, access controls, monitoring, chain-of-custody procedures, treatment resilience, and emergency communication perform during compound disruptions rather than only under normal operating conditions [125].

Climate-driven increases in environmental contamination can further complicate surveillance by raising the background frequency of foodborne and waterborne disease, increasing laboratory workloads, and making deliberate contamination more difficult to distinguish from natural events. Extreme heat and precipitation have been associated with location- and serovar-specific increases in salmonellosis, while testing after Hurricane Harvey found total coliform and *Escherichia coli* occurrence in private wells to be approximately 1.5- and 2.8-fold higher than baseline, respectively [126]. These events occur against an estimated annual U.S. burden of 48 million foodborne illnesses and 7.15 million domestically acquired waterborne illnesses, although these totals are not entirely climate attributable [127,128,129]. Preparedness should therefore combine climate and hydrologic data with epidemiology, genomic sequencing, water quality monitoring, process deviations, physical security records, traceability information, and cyber alerts. It should also include event-triggered sampling after floods, heat waves, wildfire, source-water changes, or power failures; redundant power, refrigeration, communications, laboratories, and water supplies; secure manual operating procedures; and exercises involving simultaneous natural hazard, contamination, and cyber incidents. Particular attention is required for farms, private wells, small water systems, and underserved rural, Tribal, and economically disadvantaged communities, where monitoring capacity and infrastructure redundancy are often limited [122,123,129].

## 3. Conclusions

Algal toxins should be understood as a consequential component of modern food and water defense rather than a marginal subset of environmental health. Their ability to contaminate seafood, drinking water, algal ingredients, and other water-dependent food pathways; to evade sensory detection; to resist routine preparation; and to disrupt interconnected supply systems makes them important hazards for preventive planning. The significance of this review lies in integrating algal toxin toxicology, harmful algal bloom ecology, seafood safety, drinking-water protection, preparedness for intentional adulteration, climate change, and emergency response into a single defense-oriented framework. This perspective does not imply that most algal toxin events are deliberate. Rather, it emphasizes that high-consequence toxin incidents require preparedness systems capable of functioning regardless of whether contamination is ultimately classified as natural, accidental, or intentional.

Credible preparedness, therefore, requires more than bloom awareness alone. It requires coordinated surveillance of source waters and harvest areas, risk-based testing, supplier and chain-of-custody controls, product-hold and traceback capacity, treatment resilience for drinking-water systems, clinical and poison-control recognition, public advisories, and emergency communication across the food and water continuum. As climate change, global seafood trade, expanding aquaculture, changing recreational and subsistence harvesting patterns, and water-system vulnerabilities alter exposure profiles, algal toxins provide a practical test case for whether contemporary food and water defense systems can manage biologically derived hazards that are environmentally driven, operationally complex, and socially disruptive.

## Figures and Tables

**Table 1:** Major algal toxin classes, representative toxins, source organisms, and primary molecular targets.

Toxin Class	Representative Toxins	Main Source Organisms	Primary Molecular Target/Action
Paralytic shellfish poisoning (PSP)	Saxitoxin, neosaxitoxin, gonyautoxins	Alexandrium, Gymnodinium, Pyrodinium, some cyanobacteria	Blockade of voltage-gated sodium channels [18,19,20,21]
Amnesic shellfish poisoning (ASP)	Domoic acid	Pseudo-nitzschia	Agonist at ionotropic glutamate receptors; excitotoxicity [22,23,24,25,26,27]
Diarrhetic shellfish poisoning (DSP)	Okadaic acid, dinophysistoxins	Dinophysis, Prorocentrum	Inhibition of protein phosphatases 1 and 2A [25,28]
Ciguatera fish poisoning (CFP)	Ciguatoxins, maitotoxin, palytoxin-like compounds	Gambierdiscus and related dinoflagellates	Persistent activation of voltage-gated sodium channels; related membrane disruption [29]
Neurotoxic shellfish poisoning (NSP)	Brevetoxins	*Karenia brevis*	Persistent activation of voltage-gated sodium channels [29]
Azaspiracid poisoning (AZP)	Azaspiracids	Azadinium and related dinoflagellates	Multitarget cytotoxic effects; mechanism of action remains incompletely defined [30]
Palytoxin poisoning	Palytoxin and congeners	Ostreopsis, zoanthids, and some marine organisms	Disruption of Na+/K+-ATPase [31,32]
Freshwater cyanotoxin poisoning	Microcystins, nodularin, cylindrospermopsin, anatoxin-a, guanitoxin	Freshwater cyanobacteria including Microcystis, Dolichospermum, Planktothrix, Cylindrospermopsis, and Aphanizomenon	Hepatotoxic protein phosphatase inhibition; protein-synthesis inhibition; nicotinic acetylcholine receptor agonism; acetylcholinesterase inhibition

Footnotes: This table includes both marine algal toxin classes associated with seafood poisoning syndromes and freshwater cyanobacterial toxin classes, including microcystins and related cyanotoxins. PSP, ASP, DSP, CFP, NSP, and AZP are the principal human seafood poisoning syndromes caused by marine phycotoxins. The listed source organisms are representative and not exhaustive. Molecular targets summarize the dominant toxic mechanism most relevant to human disease.

**Table 2:** Freshwater and marine algal toxins: distribution, physicochemical tendencies, and public health relevance.

Environmental Setting	Major Toxin Groups	Dominant Physicochemical Tendency *	Principal Exposure Pathway	Public Health Relevance
Marine	PSP, ASP, DSP, CFP, NSP, palytoxin-related toxins	Mixed; hydrophilic toxins often produce rapid systemic effects; lipophilic toxins show broader tissue distribution [23,24,25,32,36,70,71,72,73]	Seafood consumption; in some cases, aerosol inhalation	Shellfish and fish safety; coastal respiratory events
Freshwater	Microcystins, nodularin, cylindrospermopsin, saxitoxin, anatoxin-a, guanitoxin, dermatotoxins	Many are hydrophilic [74]; some are membrane-active irritants	Drinking water, recreation, animal ingestion, skin contact	Municipal water safety; livestock and wildlife mortality
Mixed coastal systems	Brevetoxins, ciguatoxins, palytoxin-like compounds	Often lipophilic and persistent [23,24,25,32,36,70,71,72,73]	Seafood, aerosols, marine contact	Beach-related illness; seafood advisories; fishery impacts

* From PubChem, NIH, National Library of Medicine, National Center for Biotechnology Information [https://www.niehs.nih.gov/health/topics/agents/algal-blooms], access on 20 August 2026. Footnotes: Physicochemical behavior influences tissue distribution, clearance, and persistence of toxicity. Hydrophilic toxins often act quickly and may be cleared faster, whereas lipophilic toxins may distribute more widely and persist longer. The route of exposure is a major determinant of the clinical syndrome.

**Table 3:** Analytical and regulatory approaches for algal toxins.

Approach	Use	Strengths	Limitations	Representative Application
Mouse bioassay [76,77]	Historical regulatory screening	Detects overall biological toxicity	Ethical concerns, low specificity, variability	Legacy screening for shellfish toxins
Chemical analysis [78,79,80,81,82]	Toxin identification and quantitation	High specificity, supports congener profiling	Requires standards and instrumentation	LC-MS/MS detection of marine and freshwater toxins
Immunoassays [76,83]	Screening for selected toxins	Rapid, relatively simple	May miss analogs or structurally divergent toxins	Shellfish monitoring
Receptor-binding assays [73,83]	Functional detection of bioactive toxin classes	Detects biologically active analogs	Limited to toxins with known binding targets	Saxitoxin and brevetoxin screening
Functional cell-based assays [84]	Assessment of physiological toxicity	Captures integrated cellular response	Cell-line sensitivity varies; interpretation can be complex	Neuronal or hepatocyte toxicity testing
Primary neuronal culture/MEA systems [27,85]	Mechanistic neurotoxicity assessment	Sensitive, dynamic, non-invasive electrophysiology	Technically specialized	Early detection of toxin effects on neuronal firing

**Table 4:** Human syndromes associated with algal toxins, exposure routes, and hallmark clinical findings.

Syndrome	Typical Toxins	Common Exposure Route	Hallmark Clinical Findings	Typical Clinical Concern
Paralytic shellfish poisoning [36,37]	Saxitoxins	Contaminated shellfish	Perioral numbness, paresthesia, weakness, paralysis	Respiratory failure
Amnesic shellfish poisoning [23,24,70,71,72]	Domoic acid	Shellfish, occasionally other seafood	Confusion, disorientation, amnesia, seizures	Persistent neurologic injury
Diarrhetic shellfish poisoning [25]	Okadaic acid, dinophysistoxins	Shellfish	Diarrhea, abdominal cramps, nausea, vomiting	Dehydration, transient illness
Ciguatera fish poisoning [29,66,67,68,73,87]	Ciguatoxins	Reef fish	GI symptoms, paresthesia, temperature reversal, fatigue	Prolonged neurologic symptoms
Neurotoxic shellfish poisoning [29,68]	Brevetoxins	Shellfish; aerosol near blooms	Paresthesia, dizziness, bronchial irritation, respiratory complaints	Neuro-respiratory toxicity
Cyanobacterial hepatotoxicity [88,89,90,91]	Microcystins, nodularin, cylindrospermopsin	Drinking water, recreation, food	Nausea, vomiting, abdominal pain, liver dysfunction	Hepatic injury
Cyanobacterial neurotoxicity [92,93]	Saxitoxin, anatoxin-a, guanitoxin	Drinking water, animal exposure, recreation	Tremor, weakness, fasciculations, paralysis	Respiratory compromise
Cyanobacterial dermatitis [94,95,96]	Lyngbyatoxins, aplysiatoxins	Skin contact, aerosols	Rash, erythema, blistering, irritation	Local tissue injury

Footnotes: Clinical manifestations vary with dose, route, toxin combination, and host factors. Some toxins produce overlapping syndromes, and sublethal exposures may still be clinically significant. Aerosol exposure is particularly relevant for brevetoxins and some cyanobacterial irritants.

## Data Availability

No new data were created or analyzed in this study. Data sharing is not applicable to this article.
